# A Case Report of Nonketotic Hyperglycemic Seizures: A Diagnostic Dilemma

**DOI:** 10.7759/cureus.11416

**Published:** 2020-11-10

**Authors:** Vamsi Krishna Gorijala, Likhita Shaik, Praveen Kowtha, Parneet Kaur, Venkata Sundarachary Nagarjunakonda

**Affiliations:** 1 Neurology, Guntur Medical College, Guntur, IND; 2 Cardiovascular Disease, Mayo Clinic, Rochester, USA; 3 Internal Medicine, Department of Health and Family Welfare, Government of Punjab, Chandigarh, IND; 4 Medicine, Sri Guru Ram Das Institute of Medical Sciences and Research, Amritsar, IND

**Keywords:** hyperglycemic seizures, epilepsia partialis continua, nonketotic hyperglycemia, nkh-induced seizures

## Abstract

Nonketotic hyperglycemia (NKH) is a rare but serious complication of uncontrolled diabetes mellitus that occurs acutely with a mortality rate of more than 50%. This condition presents with a clinical syndrome consisting of profound hyperglycemia, hyperosmolality, and dehydration. Infrequently, the patients also present with seizure activity. The most common types of seizures observed in this condition are focal seizures, as opposed to the generalized seizures observed in hypoglycemia-induced seizures. Though various hypotheses tried to explain NKH-induced seizure activity, the actual mechanism remains unknown. The treatment modalities include the management of hyperglycemia and circulatory collapse. However, the role of anti-epileptics is controversial. We herein illustrate an atypical case of focal faciobrachial seizures in a young female patient, which occurred as a rare complication of NKH.

A 21-year-old female was admitted with multiple jerking and spasmodic movements of the right upper limb and face, with no significant neurological findings. Past medical history was significant for uncontrolled type 2 diabetes mellitus and multiple episodes of focal seizures. On laboratory examination, serum osmolarity was 309 mOsm/L, blood glucose was 364 mg/dL, HbA1c was 12.1%, and ketone bodies were absent. MRI brain showed large subtle T2 FLAIR (T2-weighted fluid-attenuated inversion recovery) cortical hyperintensities in the left frontal, temporal, parietal, and occipital regions with subcortical hypointense areas. The EEG illustrated a background slowing and generalized spikes, polyspikes, and sharp-wave discharges with post-ictal slowing. The patient's seizures were initially refractory to insulin therapy and resolved with the use of dual anti-epileptics.

Thus, to conclude, our case represents a diagnostic dilemma with MRI findings pointing towards NKH as the underlying etiology of focal seizures, with the resolution of seizures only occurring with the addition of anti-epileptics to insulin therapy.

## Introduction

Diabetes mellitus can cause many neurological complications such as peripheral neuropathy, autonomic neuropathy, neuropathic osteoarthropathy, and encephalopathy. Hyperglycemic seizures are considered one of the most dangerous neurological complications of diabetes mellitus (both type I and type II) [1,2]. Seizures due to nonketotic hyperglycemia (NKH) are more often seen in people >50 years of age and are relatively rare in young adults and children. It has a female-to-male ratio of 1.33:1 [3]. Hyperglycemic seizures represent a clinical condition with high blood glucose levels, normal or increased serum osmolality, and negative urine ketone bodies [4]. However, hyperglycemia is not the only causative factor for seizures. Factors such as genetic predisposition, hydration status, comorbidities, and other health conditions also play a role [5]. Management of hyperglycemia is the most important initial step. The role of anti-epileptics in the management of hyperglycemic seizures is unclear [4]. 

We present a case of focal seizures as a rare complication of NKH, which was refractory to anti-hyperglycemic treatment, but the seizures subsided with anti-epileptics.

## Case presentation

A 21-year-old right-hand-dominant female with a history of uncontrolled type 2 diabetes mellitus presented to the emergency department with multiple episodes (>10) of jerking and spasmodic movements of the right upper limb and right side of the face lasting approximately 1 minute, which started the previous day. These movements were associated with tongue biting and involuntary passage of urine. She remained confused for about 5 minutes after each episode before regaining consciousness. History was negative for nausea, vomiting, fever, headache, loss of consciousness, and sensory and vision changes. 

She was diagnosed with type 2 diabetes mellitus about one year ago and was using metformin 500 mg and glimepiride 1 mg. She had many similar seizure episodes in the past 10 months. The patient’s history suggests a non-compliance with her medications. She had no known neurological abnormalities.

On physical examination, she was alert, conscious, and well oriented to time, place, and person. Her speech was incoherent. Her Glasgow Coma Scale (GCS) was E_4_V_4_M_6_. Kernig's and Brudzinski's signs were negative. Cranial nerve, motor, and sensory examinations were normal. Vitals were stable. Multiple brief episodes of right-sided focal faciobrachial seizures were observed after admission.

Investigations at admission showed a blood glucose of 364 mg/dL (normal range: 80-140 mg/dL) and serum osmolarity was 309 mOsm/L (normal range: 285-295 mOsm/L). Ketone bodies were absent. Her HbA1C was 12.1% (good diabetic control range: 6.1-7%). Electrolytes such as serum electrolytes were within normal limits. Her blood urea nitrogen level was 16 mg/dL (normal range: 8-21 mg/dL) and serum creatinine was 0.6 mg/dL (normal range: 0.5-1.1 mg/dL). The viral markers for HIV, hepatitis B, and hepatitis C were negative.

An immediate intravenous infusion of regular insulin was started as per the standard guidelines, which was later substituted with subcutaneous insulin. Non-contrast computed tomography (CT) scan of the brain was normal. The seizures were not controlled at this point and kept frequently occurring, with each episode lasting approximately a minute.

The patient developed a terminal neck stiffness the next day. Cerebrospinal fluid (CSF) analysis was ordered to rule out meningitis, and it showed clear fluid and absence of xanthochromia. The total WBC count was 3 cells/mm^3^ (normal range: 0-5 cells/mm^3^). The differential count was 100% lymphocytes and 0% PMNs (polymorphonuclear leukocytes). CSF glucose level was 72 mg/dL (normal range: 30-90 mg/dL) and CSF protein was 31 mg/dL (normal range: 15-45 mg/dL).

Since the seizure activity did not abate with insulin therapy even after normalizing the glucose levels, anti-epileptics were considered. The patient received an oral dose of carbamazepine (200 mg). The number of subsequent seizure episodes reduced but was not completely terminated. Hence, a second anti-epileptic, oral clobazam (10 mg once daily), was added. The seizures were finally controlled after 72 hours, and the patient remained seizure-free thereafter. 

**Figure 1:**
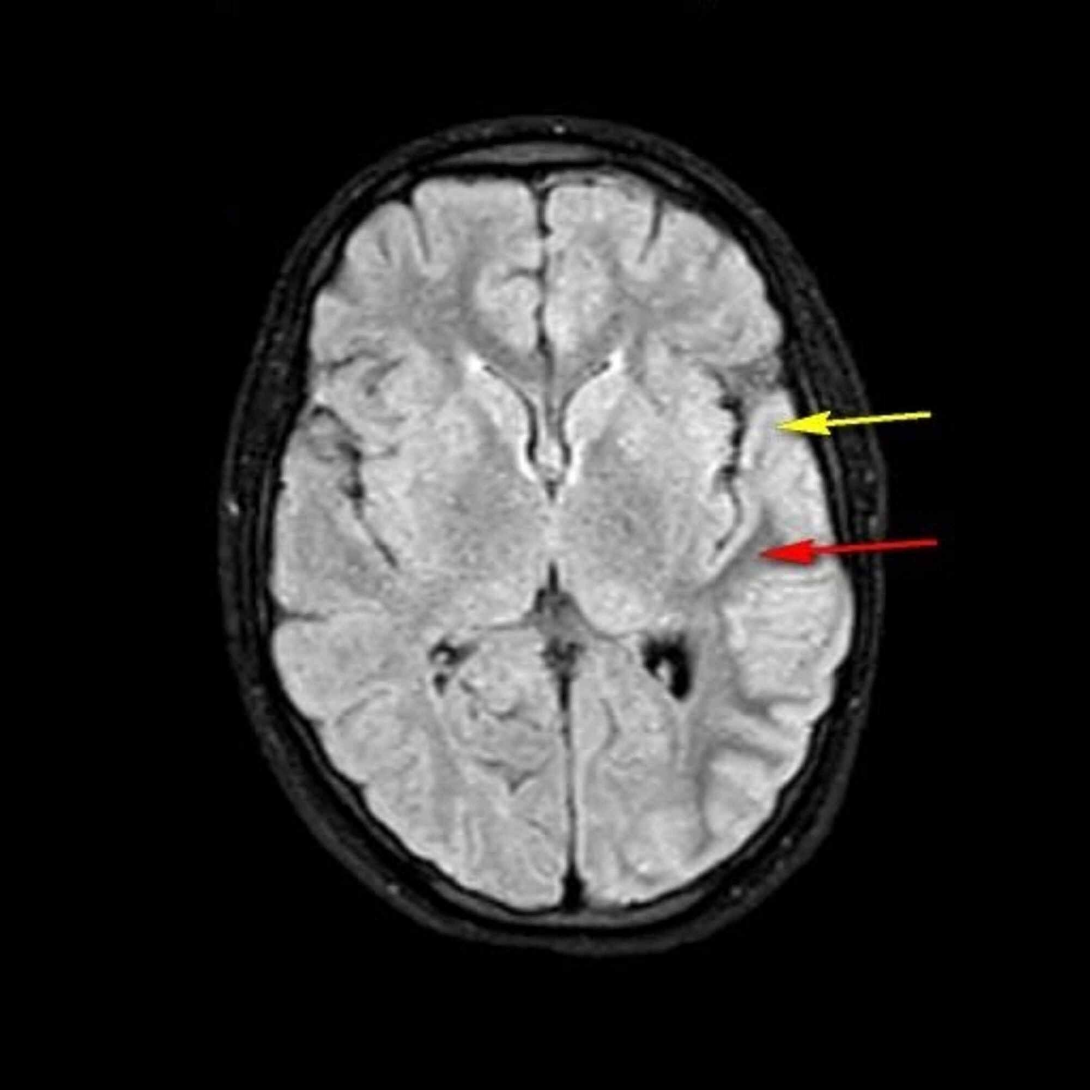
MRI of the brain: T2-FLAIR sequence The T2-FLAIR image of the patient is shown. The red arrow indicates subcortical hypointensity, and the yellow arrow points to the overlying cortical hyperintensity, which are the classical MRI findings to be expected in NKH-induced seizures. NKH, nonketotic hyperglycemia; T2-FLAIR, T2-weighted fluid-attenuated inversion recovery

**Figure 2:**
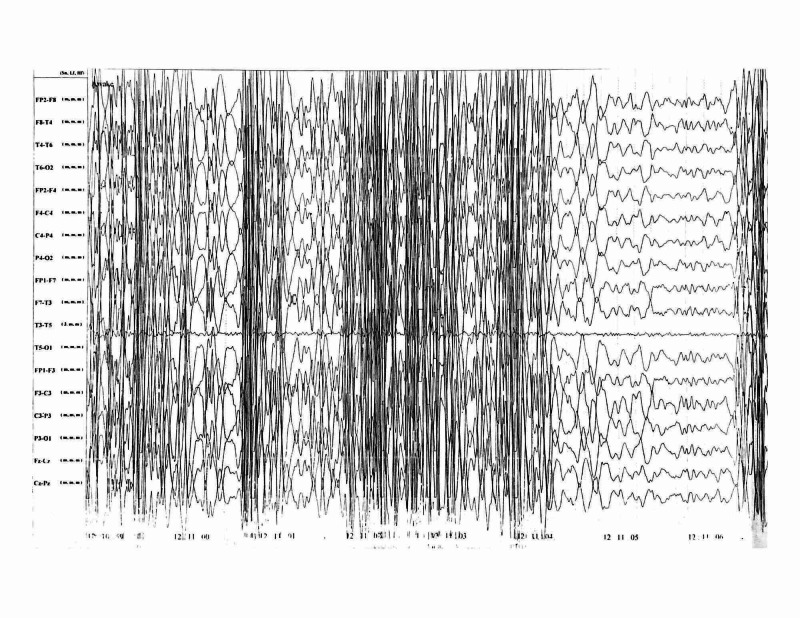
Awake EEG of the patient The awake EEG of the patient showing generalized spikes, polyspikes, and sharp-wave discharges, with post-ictal slowing. This may be due to the secondary generalization of the seizure. EEG, electroencephalography

Magnetic resonance imaging (MRI) of the brain showed large subtle T2-FLAIR (T2-weighted fluid-attenuated inversion recovery) cortical hyperintensities in the left frontal, temporal, occipital, and parietal regions with subcortical hypointense areas (Figure 1). The electroencephalography (EEG) showed a background slowing and generalized spikes, polyspikes, and sharp-wave discharges with post-ictal slowing (Figure 2).

After seven days of admission, she was discharged as she was stable on the same treatment regime, and no additional seizure episodes were observed. The patient received counseling on medication compliance, and regular follow-ups were scheduled.

## Discussion

Diabetes and prediabetes affect more than 100 million adults living in the United States [6]. It is estimated that the deranged blood glucose levels occurring in diabetes have been associated with various chronic neurological complications that start as sensory and motor neuropathy or dysautonomia [7] and progress into cognitive dysfunction, dementia, or depression. Acute complications such as nausea, vomiting, dysphagia, headaches, strokes, loss of consciousness, and seizures occur frequently. Among them, seizures are known to have high rates of morbidity and mortality [8-10]. Hence, focusing on the early recognition of the presenting type of seizures, its cause and treatment are of utmost importance in light of the near future estimation that by 2050, one in three Americans will meet the diagnostic criteria for diabetes and prediabetes based on the blood sugar levels [11,12].

Around 25% of diabetic patients experience seizures and are frequently related to the electrolyte (mainly sodium and potassium) and osmolality disturbances such as uremia and ketoacidosis. Multiple other mechanisms such as local brain damage, microvascular lesions in the brain, metabolic factors, and gene mutations also contribute to this condition [13,14]. Various factors such as infection, surgery, dialysis, tube feedings, and stress are a few known precipitating causes of the event. The absence of any such factors poses a challenging question in our case [15].

The literature suggests that NKH is a rare cause of seizures in diabetics compared to the more frequently occurring ketoacidosis-induced seizures [16]. NKH-induced seizures are known to occur in adults for more than 50 years. The occurrence in younger patients has been reported in very scant numbers [17]. Here we presented such a case of NKH-induced seizures in a 21-year-old female patient.

NKH can be differentiated by their characteristic of being focal in nature as compared to the more generalized appearance of hypoglycemia-induced seizures [18]. Identifying such subtle differences in diagnosing NKH is a key to the early treatment of the condition considering its refractory nature to the treatment with the traditional anti-epileptic therapy. However, it is more challenging to manage a patient with an atypical presentation like our patient, who presented with focal seizures with secondary generalization. In such cases, all measures, such as a trial of anti-epileptics, are necessary to terminate the seizures, which have grave outcomes if untreated [2,18].

The diagnosis of NKH entails the need for imaging modalities such as CT scan or MRI. Typical imaging results such as focal subcortical T2 hypointensity in the cerebral hemispheres, gyral swelling, contrast retention in the surrounding meninges, and diffusion restriction clarify the diagnosis and aid in managing an NKH condition [19]. However, our patient's clinical picture poses a diagnostic dilemma in light of a similar history of focal seizures. Features of seizures related to a seizure disorder and seizures related to diabetes are key differentials that needed to be clearly distinguished, owing to the different treatment goals. In a nonketotic state with imaging evidence pointing toward NKH, our treatment focused on treating the cause, i.e., hyperglycemia. However, the typical nature of NKH-induced seizures being refractory to the traditional anti-epileptics achieved stability in our case only after the initiation of dual anti-epileptic therapy. We believe that a previous history of similar episodes could be a sign of impending NKH-induced seizure activity. Though several causes such as local brain damage, microvascular brain lesions, immunity-related issues, gene mutations, and metabolic factors have been suggested for NKH-induced seizures, more research is warranted to clarify the mechanisms that lead to such atypical presentations, as seen in our patient [13].

## Conclusions

Hyperglycemia remains one of the least common causes of seizures to this day. The presence of high blood glucose, absence of Ketone bodies in urine, and normal serum osmolality are the most common findings. Some patients may not even have a history of diabetes mellitus. Although various hypotheses try to explain the cause of NKH-induced seizures, the exact mechanism of these seizures is unknown. Brain MRI and EEG are the most valuable diagnostic options. It is believed that the mainstay of treatment depends on normalizing glucose levels rather than anti-epileptics. However, in our case, the addition of dual anti-epileptic therapy terminated the seizures, which were uncontrolled even after achieving satisfactory glycemic control with insulin. As there is a lot to be studied about the pathogenesis and the different treatment options for NKH-induced seizures, further research into this condition is warranted.

## References

[1] Maccario M (1968). Neurological dysfunction associated with nonketotic hyperglycemia. Arch Neurol.

[2] Tiamkao S, Pratipanawatr T, Tiamkao S, Nitinavakarn B, Chotmongkol V, Jitpimolmard S (2003). Seizures in nonketotic hyperglycemia. Seizure.

[3] Scherer C (2005). Crises epileptiques révélatrices d'une hyperglycémie sans cétose [Seizures and non-ketotic hyperglycemia]. Presse Med.

[4] Wang Wang, X X (2017). Nonketotic hyperglycemia-related epileptic seizures. Chin Neurosurg.

[5] Huang CW, Tsai JJ, Ou HY, Wang ST, Cheng JT, Wu SN, Huang CC (2008). Diabetic hyperglycemia is associated with the severity of epileptic seizures in adults. Epilepsy Res.

[6] (2020). New CDC report: more than 100 million Americans have diabetes or prediabetes. https://www.cdc.gov/media/releases/2017/p0718-diabetes-report.html.

[7] Whitsell LJ (1962). Neurologic complications of diabetes. Calif Med.

[8] Seaquist ER (2015). The impact of diabetes on cerebral structure and function. Psychosom Med.

[9] Stahlman GC, Auerbach PS, Strickland WG (1988). Neurologic manifestations of non-ketotic hyperglycemia. J Tenn Med Assoc.

[10] Chung SJ, Lee JH, Lee SA, No YJ, Im JH, Lee MC (2005). Co-occurrence of seizure and chorea in a patient with nonketotic hyperglycemia. Eur Neurol.

[11] (2020). National Diabetes Statistics Report, 2020. https://www.cdc.gov/diabetes/library/features/diabetes-stat-report.html.

[12] Boyle JP, Thompson TJ, Gregg EW, Barker LE, Williamson DF (2010). Projection of the year 2050 burden of diabetes in the US adult population: dynamic modeling of incidence, mortality, and prediabetes prevalence. Popul Health Metr.

[13] Yun C, Xuefeng W (2013). Association between seizures and diabetes mellitus: a comprehensive review of literature. Curr Diabetes Rev.

[14] Arieff AI, Doerner T, Zelig H, Massry SG (1974). Mechanisms of seizures and coma in hypoglycemia. Evidence for a direct effect of insulin on electrolyte transport in brain. J Clin Invest.

[15] (2020). Nonketotic Hyperglycemia. https://www.epilepsy.com/living-epilepsy/epilepsy-and/professional-health-care-providers/co-existing-disorders/metabolic-5.

[16] Maccario M, Messis CP, Vastola EF (1965). Focal seizures as a manifestation of hyperglycemia without ketoacidosis. A report of seven cases with review of the literature.. Neurology.

[17] Moien-Afshari F, Téllez-Zenteno JF (2009). Occipital seizures induced by hyperglycemia: a case report and review of literature. Seizure.

[18] Monami M, Mannucci E, Breschi A, Marchionni N (2005). Seizures as the only clinical manifestation of reactive hypoglycemia: a case report. J Endocrinol Invest.

[19] Sasaki F, Kawajiri S, Nakajima S (2016). Occipital lobe seizures and subcortical T2 and T2* hypointensity associated with nonketotic hyperglycemia: a case report. J Med Case Rep.

